# Advanced integration of 2DCNN-GRU model for accurate identification of shockable life-threatening cardiac arrhythmias: a deep learning approach

**DOI:** 10.3389/fphys.2024.1429161

**Published:** 2024-07-12

**Authors:** Abduljabbar S. Ba Mahel, Shenghong Cao, Kaixuan Zhang, Samia Allaoua Chelloug, Rana Alnashwan, Mohammed Saleh Ali Muthanna

**Affiliations:** ^1^ School of Life Science and Technology, University of Electronic Science and Technology of China, Chengdu, China; ^2^ Department of Information Technology, College of Computer and Information Sciences, Princess Nourah bint Abdulrahman University, Riyadh, Saudi Arabia; ^3^ Institute of Computer Technologies and Information Security, Southern Federal University, Taganrog, Russia

**Keywords:** dangerous arrhythmias, recognition, deep learning networks, data synthesis, scalogram

## Abstract

Cardiovascular diseases remain one of the main threats to human health, significantly affecting the quality and life expectancy. Effective and prompt recognition of these diseases is crucial. This research aims to develop an effective novel hybrid method for automatically detecting dangerous arrhythmias based on cardiac patients’ short electrocardiogram (ECG) fragments. This study suggests using a continuous wavelet transform (CWT) to convert ECG signals into images (scalograms) and examining the task of categorizing short 2-s segments of ECG signals into four groups of dangerous arrhythmias that are shockable, including ventricular flutter (C1), ventricular fibrillation (C2), ventricular tachycardia torsade de pointes (C3), and high-rate ventricular tachycardia (C4). We propose developing a novel hybrid neural network with a deep learning architecture to classify dangerous arrhythmias. This work utilizes actual electrocardiogram (ECG) data obtained from the PhysioNet database, alongside artificially generated ECG data produced by the Synthetic Minority Over-sampling Technique (SMOTE) approach, to address the issue of imbalanced class distribution for obtaining an accuracy-trained model. Experimental results demonstrate that the proposed approach achieves high accuracy, sensitivity, specificity, precision, and an F1-score of 97.75%, 97.75%, 99.25%, 97.75%, and 97.75%, respectively, in classifying all the four shockable classes of arrhythmias and are superior to traditional methods. Our work possesses significant clinical value in real-life scenarios since it has the potential to significantly enhance the diagnosis and treatment of life-threatening arrhythmias in individuals with cardiac disease. Furthermore, our model also has demonstrated adaptability and generality for two other datasets.

## 1 Introduction

Cardiovascular disease remains one of the most severe threats to human health, with a significant impact on quality of life and longevity. Within the framework of research in this area, one of the most urgent tasks is the classification of arrhythmias (Zhang et al., 2022) since the effective and accurate identification of types of arrhythmias is a crucial aspect in making decisions about treating and managing heart diseases. Among the variety of arrhythmias, special attention is paid to ventricular flutter (VFL), ventricular fibrillation (VF) (Zeppenfeld et al., 2022), ventricular tachycardia (VTTdP), and high-rate ventricular tachycardia (VTHR), as they are characterized by a high degree of severity and require immediate medical attention (Rajendra Acharya et al., 2018). Arrhythmias are deviations from the heart’s normal rhythm and can range from mild, almost imperceptible changes to life-threatening conditions. One critical challenge cardiologists and heart specialists face is the accurate classification of arrhythmia types to determine the best treatment strategy. In addition, there are restrictions on the time of the ECG analysis, which in different studies varies from 2 to 10 s (Bukhari et al., 2023). Reducing the analysis time seems extremely important since the instant indication of a dangerous violation, especially in implanted cardioverter-defibrillators (CDs), helps the patient save life. With the availability of large volumes of electrocardiogram (ECG) data and the development of machine learning technologies, it has become possible to classify arrhythmias with high accuracy (Xiao et al., 2023) automatically. Machine learning algorithms such as neural networks and signal processing algorithms can analyze ECG data to determine the type of arrhythmia accurately. There are a lot of algorithms for the automatic detection of cardiac disorders based on ECG, which are based on the detection of the ventricular ECG complex wave (QRS complex) and the analysis of the morphological features of this complex (Li et al., 1995; Al-Naima and Al-Timemy, 2009; Pandit et al., 2017). This method is appropriate for exploring dangerous arrhythmias, as the QRS complex is a pivotal indicator of the heart’s state and electrical activity. Many works based on the isolation of cardio cycles based on signal segmentation, which includes the detection of PQRS-T waves. The detection of QRS complexes in ECG signals has been carried out for many years with the help of widely used methods such as the pattern matching method in fetal ECG analysis (Liu et al., 2019), the differential threshold method (Pandit et al., 2017), and wavelet transform (Tuncer et al., 2019). Some algorithms have also been developed to extract features from P and T waves (Madeiro et al., 2017). RR intervals (RRI) are one of the most essential ECG features used for ECG classification (Kennedy et al., 2016). In addition, morphological features such as wave amplitude and ECG wave intervals were used, such as morphological features obtained from P-QRS-T waves of ECG signals (Alquraan et al., 2019). Some other features can be obtained using ECG signal processing methods, such as higher-order spectral cumulants (Alquraan et al., 2019), discrete and continuous wavelet transforms, and independent component analysis. However, some of the above methods have disadvantages, such as dependence on the subjective perceptions of the subjects, variability of results depending on the instructions given to the subject, and the requirement of enormous computing resources to analyze extensive data. Therefore, to diagnose high-risk arrhythmias correctly, it is necessary to consider other technologies, such as deep learning, that can extract unique characteristics of the signals by end-to-end form, etc. Many deep learning neural network models have been used to analyze ECG signals in recent years due to their high efficiency, such as convolutional neural networks (CNN) (Byeon et al., 2019; Olanrewaju et al., 2021; Xiao et al., 2023; Ba Mahel and Kalinichenko, 2024). In terms of the model input method, both one-dimensional fragments of the initial time readings of the ECG signal (1D-CNN) (Rajendra Acharya et al., 2018; Acharya et al., 2019) and two-dimensional representations of time fragments (2D-CNN) (Byeon et al., 2019; Olanrewaju et al., 2021) are used. The 2D conversion of an ECG signal to an image is related to the tremendous success of applying deep neural networks in image analysis. Short-time Fourier transform (STFT) - spectrograms (Al-Naima and Al-Timemy, 2009), continuous wavelet transform (CWT) - scalograms (Byeon et al., 2019; Olanrewaju et al., 2021), and Markov transition fields (MTF) are commonly used methods conversing a one-dimensional signal into a two-dimensional image. However, it is worth noting that the deep learning model also faces particular challenges in arrhythmia classification. Lack of data, irregularity in the distribution of arrhythmia types, and noise in the signals can affect the accuracy and reliability of the classification. It requires careful preparation of the data and the development of algorithms capable of handling such complexities. Recent works using continuous wavelet transform (CWT)—scalograms and convolutional neural networks (CNN) are the most closely related to the subject under consideration. Using the CNN model, Acharya et al. (2018) proposed a new tool for automatically differentiating shockable and non-shockable ventricular arrhythmias. The authors processed 2-s ECG fragments with an eleven-layer CNN model to identify life-threatening ventricular arrhythmias. Their work demonstrated the effectiveness of the proposed approach in accurately detecting shock and non-shock ventricular arrhythmias using ECG signals, providing a promising tool for the early diagnosis and treatment of life-threatening ventricular arrhythmias (Rajendra Acharya et al., 2018). The maximum accuracy obtained by the authors was 93.18%. The shortcomings of this work are that training requires a considerable dataset, the classification is binary, and the performance indexes of the proposed CNN model require improvements. Olanrewaju et al. (2021) developed an integrated model using CWT and deep neural networks to accurately classify ECG signals to detect arrhythmia, congestive heart failure, and normal sinus rhythm. Their work demonstrated the effectiveness of the proposed approach in accurately predicting common heart disease using ECG signals, providing a promising method for diagnosing heart disease. Byeon et al. (2019) compared applying deep machine learning models in biometrics using ECG scalograms. The authors proposed a biometric recognition system that used ECG waveforms and deep learning models to achieve a high accuracy of 94% in biometric recognition. Their results showed that the proposed method outperformed conventional ECG-based biometric recognition methods, demonstrating the effectiveness of the proposed approach. Wang et al. (2021) proposed an automatic ECG classification method that uses CWT and CNN. The method has achieved an overall performance of 67.47% and 68.76% sensitivity and F1-score, respectively, in the classification of ECG signals of the following class: Normal (N), Ventricular Ectopic Beat (VEB), Supraventricular Ectopic Beat (SVEB), and Fusion Beat (F), demonstrating the effectiveness of the proposed approach. However, the overall performance achieved by this approach still has to be improved. Ba Mahel et al. (2022) proposed an arrhythmia classification method that uses scalograms of heart vector magnitude (HVM), signal segmentation, and a deep network to classify five different classes of arrhythmias (healthy control (HC), myocardial infarction (MI), cardiomyopathy (CM), bundle branch block (BB) and dysrhythmias (DS)), achieving a high classification accuracy of 98%. The proposed approach has demonstrated the potential of using deep learning methods for accurate ECG classification. However, this study uses the HVM for arrhythmia classification, which has limited information since HVM reduces the electrical activity of the heart to a vector representation, potentially losing some vital information that can be useful for accurately classifying arrhythmias. Other characteristics of the ECG, such as ECG waveform and duration, may be more informative.

In (Ba Mahel and Kalinichenko, 2022), the same author presented a practical algorithm for classifying cardiac cycles based on images using a convolutional neural network. However, it is essential to note that using only two classes in the work may not be sufficient for addressing real-life problems. Moreover, the F1-score (73.1%), recall (85.4%), and precision (68.6%) all have the potential for further improvement. This study utilizes CWT technology to convert 2-s ECG fragments into scalograms, followed by developing a novel lightweight hybrid neural network that combines a 2DCNN and a Gated Recurrent Unit (GRU). The objective of developing this network is to accurately categorize four shockable types of dangerous arrhythmias on short 2-s fragments of ECG signals. The results of the conducted experiments indicate that the average classification accuracy, F1-score, specificity, and sensitivity for all classes were 97.75%, 97.75%, 99.25%, and 97.75%, respectively. These findings significantly improve compared to existing approaches and effectively address the constraints identified in earlier research studies. The contributions of this manuscript are summarized as follows:

(1) Our study makes a significant contribution to the field of medical diagnostics by developing a novel lightweight hybrid model to improve the classification of arrhythmias on short ECG signals.
(2) The application of this model to the classification of shockable arrhythmia effectively utilizes a combination of wavelet transform, 2DCNN, and GRU.
(3) Using synthetic data generated by the Synthetic Minority Over-sampling Technique (SMOTE) method for class balancing and subsequent training of convolutional neural networks (CNNs) improves the deep learning model robustness, a prevalent concern in medical and other applications. It is particularly significant in arrhythmia classification, as it directly influences the dependability and consistency of the classification outcomes.
(4) Our experiments also contributed to deep learning methodology by providing a comparative analysis between six different state-of-the-art convolutional neural networks (CNN) in the context of data analysis. This analysis may be helpful for other researchers working in signal processing and medical data analysis to select the appropriate model for their tasks. Thus, our study has methodological implications by expanding the understanding of the capabilities of deep learning in the medical field, especially in ECG arrhythmias analysis and classification.
(5) Development of an innovative end-to-end lightweight hybrid model that is an efficient tool suitable for adaptation and application in various image classification problems.

## 2 Materials and methods

### 2.1 Real and synthetic data

This study utilized the ECG Fragment Database for the Exploration of Dangerous Arrhythmia (EFEDA, https://physionet.org/content/ecg-fragment-high-risk-label/1.0.0/, which consists of high-risk segments of ECG that were available on the PhysioNet platform (Nemirko et al., 2022). This database comprises an extensive collection of medical data primarily focused on high-risk arrhythmias. The analysis of these high-risk ECG fragments allows us to more accurately study the characteristics of various types of arrhythmias and develop algorithms that can determine them with a high degree of accuracy. Thus, this study selected the actual ECG data of VFL (C1), VF(C2), VTTdP(C3), and VTHR (C4) in this database. The quantitative composition of the selected arrhythmias is presented in Table 1.

**TABLE 1:** Information on real and synthetic ECG data.

Data	Real (Number of fragments in each class)	Synthesized (Number of fragments in each class)	Total
Class			
**VFL(С1)**	97	903	1,000
**VF(С2)**	240	760	1,000
**VTTdP(С3)**	72	928	1,000
**VTHR(С4)**	169	831	1,000
**Total**	578	3,422	4,000

Table 1 illustrates that the sample numbers among C1-C4 are pretty unbalanced. To balance our dataset, we employed synthetic data created by the SMOTE method (Chawla et al., 2002) and fragments from the ECG database. The SMOTE approach was proposed by Chawla et al., 2002. This method generates synthetic minority class samples by interpolating between existing samples, thus increasing the minority class’s representation in the dataset. This strategy is very beneficial when dealing with imbalanced datasets with significantly fewer samples in certain classes than others. For example, SMOTE has been found to improve the performance of machine learning models on imbalanced datasets (Joloudari et al., 2023). It has been used for a range of tasks, such as fraud detection (Almhaithawi et al., 2020), medical diagnostics (Lee and Lee, 2023), and credit risk assessments (Niu et al., 2020). Thus, we can increase the model’s ability to distinguish minority classes by generating synthetic data, resulting in a more balanced classification performance. The proportion of actual and synthetic data for each class is described in Table 1.

### 2.2 Transforming ECGs into scalograms

The ECG signal is a time-varying signal that depicts the heart’s electrical activity. It comprises three components: the P-wave, the QRS complex, and the T-wave. These components differ in frequency, composition, and length, which is significant for diagnosing various cardiac disorders. The ECG signal can be decomposed into its frequency components using CWT, which can ascertain the frequency composition of a signal over multiple temporal scales. It is beneficial for identifying and assessing the different elements of an ECG signal, including the P-wave, QRS complex, and T-wave. By employing CWT to transfer the ECG signal from the time domain to the time-frequency domain, we can gain a more comprehensive understanding of the underlying physiological systems responsible for generating the signal (Byeon et al., 2019; Olanrewaju et al., 2021). It can aid in diagnosing cardiovascular disease and offer crucial insights into the mechanics of electrical activity in the heart.

Transforming ECGs into scalograms offers the following advantages: depiction of localized resolution in the frequency domain, identification of momentary occurrences and subtle variations, flexibility in accommodating frequency fluctuations, examination of non-linear dynamic attributes, exploration of the integration of time-frequency properties, and avoidance of windowing issues encountered in methods like STFT (Al-Naima and Al-Timemy, 2009). In general, CWT provides a more flexible and informative approach to the analysis of ECG signals, enriching the interpretation and expanding the possibilities of diagnosing and monitoring the condition of the heart. Therefore, we transform ECGs into scalograms by CWT.

The CWT mathematical formulation (Ozaltin and Yeniay, 2023) of any signal $f\left(t\right)$ is presented in Eq. 1.

$$CWT\left(t\right)=\frac{1}{\sqrt{a}}\int_{-\infty }^{+\infty }f\left(t\right)\cdot \psi \left(\frac{t-b}{a}\right)dt$$ (1)

Where $f\left(t\right)$ is the signal, *a* is the scale parameter, b∈R is the shift parameter, and $\psi \left(t\right)$ is the mother wavelet function. We select the Morlet mother wavelet function as it has equal variance in time and frequency to perform the transformation from ECGs to scalograms, as shown in Eq. 2 (Lee and Choi, 2019):

$$\psi_{Morl}\left(t\right)=e^{2\pi it}e^{-\frac{t^{2}}{2\sigma^{2}}}=\left(\cos2\pi t+i\;\sin2\pi t\right)e^{-\frac{t^{2}}{2\sigma^{2}}}$$ (2)

The results of the CWT are many wavelet–coefficients that are the function of the scale a and shift b. In this study, we used the CWT coefficients in the form of scalograms, which can serve as input (Ba Mahel et al., 2022) into our hybrid deep neural network model to classify dangerous arrhythmias. The size of the scalograms used as input for the proposed model is 227 × 227 pixels with three color channels, which is in line with the requirements of the developed hybrid model.

### 2.3 Deep models applied for the task of classification and recognition

#### 2.3.1 2D convolutional neural network

Modern image classification problems widely use deep learning methods, especially convolutional neural networks (CNN). Convolutional Neural Networks (CNNs) provide the ability to extract distinctive characteristics from images and dynamically adjust to variations in illumination, rotations, scales, and other influencing factors. A prevalent variant of CNNs is the two-dimensional CNNs (2DCNNs), which process images represented as pixel matrices. Deep two-dimensional convolutional neural networks are composed of multiple layers, including a convolutional layer, pooling layer, activation layer, and fully connected layer. The convolutional layer applies filters to the input image and produces feature maps. The pooling layer reduces the dimensionality of feature maps and increases their invariance. The activation layer adds nonlinearity to the output of the convolutional or pooling layer. The fully connected layer performs classification based on the extracted features. 2DCNNs have several advantages over other types of CNNs, such as three-dimensional CNNs (3DCNNs). First, 2DCNNs have fewer parameters and require fewer computational resources. Secondly, 2DCNNs are more accessible to train and optimize since they avoid the problem of overfitting and gradient decay. Third, 2DCNNs can effectively deal with various image domains, such as natural, satellite, medical, etc. In recent years, many 2DCNN models that use different architectures have been used for image classification and object detection, demonstrating high accuracy and speed (Ahmad et al., 2021; Duseja, 2021; Al-gaashani et al., 2022; Kanwal and Chandrasekaran, 2022; Singh and Kumar, 2022; Tang, 2022; Al-Gaashani et al., 2023; Ashurov et al., 2023; Farhan et al., 2023; Farhan and Yang, 2023). Recently, 2DCNNs have become a vital tool in ECG analysis. For example, the work (Yousuf et al., 2023) presented an innovative approach to detecting myocardial infarction. At the same time, the study’s authors (Mewada, 2023) opened new horizons in ECG classification by proposing a computer diagnostic system based on 2DCNN. Additionally, in this research (Ayatollahi et al., 2023), the authors demonstrated the use of transfer learning to adapt 2DCNN for obstructive sleep apnea (OSA) classification. All these studies highlight the importance and effectiveness of 2DCNN in medical diagnostics.

#### 2.3.2 Gated recurrent unit (GRU) module

A Gated Recurrent Unit (GRU) is a recurrent neural network introduced by Cho et al. (Cho et al., 2014). GRU is similar to long short-term memory (LSTM) but has only two gates - reset and update. The update gate in the GRU model plays a crucial role in determining the amount of information from the past that needs to be transferred to the future. It is crucial for capturing long-term dependencies and determining what information should be stored in the model’s memory. On the other hand, the reset gate determines how much past information should be forgotten. It allows the model to estimate the importance of each input to the current state, which is helpful for prediction. The operations taking place inside the GRU can be represented by the following Eqs 3–6:

• **Update Gate**

$$z_{t}=\sigma \left(W_{z}\cdot \left[h_{t-1},x_{t}\right]\right)$$ (3)

• **Reset Gate**

$$r_{t}=\sigma \left(W_{r}\cdot \left[h_{t-1},{\;x}_{t}\right]\right)$$ (4)

• **Candidate Hidden State**

$${\tilde{h}}_{t}=\tanh\left(W\cdot \left[r_{t}⊙h_{t-1},x_{t}\right]\right)$$ (5)

• **Final Hidden State**

$$h_{t}=\left(1\;-\;z_{t}\right)⊙h_{t-1}+z_{t}⊙{\tilde{h}}_{t}$$ (6)

**FIGURE 1:**
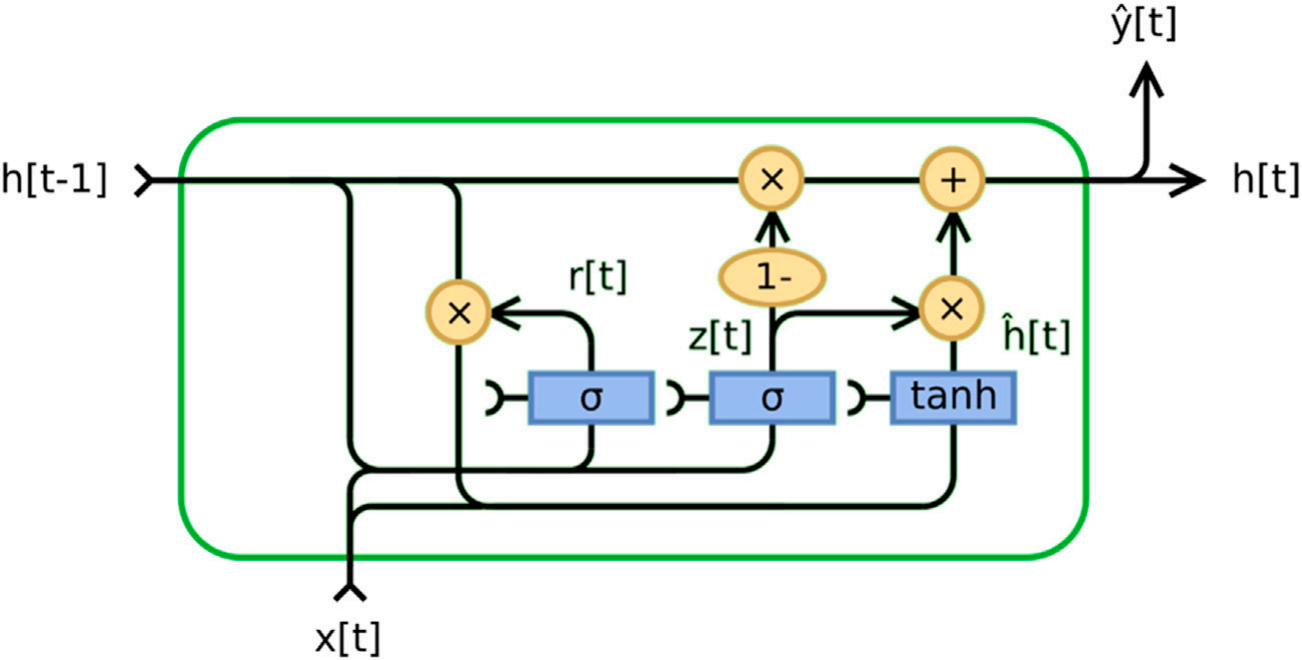
Gated recurrent unit.

In this context, σ represents the sigmoid function, tanh is the hyperbolic tangent function, $W_{z}$ , $W_{r}$ and W serve as parameter matrices, $h_{t-1}$ denotes the previous hidden state, $x_{t}$ indicates the current input, the symbol ⊙ symbolizes element-wise multiplication, and $h_{t}$ reflects the current hidden state. GRU has a lower parameter count, generally making it simpler and quicker to train than LSTM models. The architecture in Figure 1 illustrates the structure of the GRU model in the context of deep learning.

In recent years, using the GRU model for electrocardiogram (ECG) analysis has become an essential trend in the medical field. GRU, a new recurrent neural network (RNN), performs well in applications with long sequences. It can achieve a better feature extraction effect while saving computation and is very suitable for long-time series such as ECG signals (Nath et al., 2021; Yao et al., 2021).

#### 2.3.3 Description and architecture of the proposed 2DCNN-GRU model

This section provides a theoretical justification for the high accuracy, efficiency, and robustness of combining 2DCNNs with GRUs. Since 2DCNNs can process and store spatial information locally, they effectively process two-dimensional input, including images (Wang and Hu, 2021). They are perfect for processing images and other two-dimensional data because they can recognize intricate patterns and structures in data (Wang and Hu, 2021). Conversely, recurrent neural networks (RNNs) with GRUs effectively process sequential input, such as text or time series (Chen et al., 2022). They are perfect for processing sequential data because they can recall and apply knowledge from previous states to create a current prediction (Chen et al., 2022). Combining 2DCNN and GRU allows us to take advantage of both architectures (Gupta et al., 2023). 2DCNN can be used to learn spatial patterns in data, while GRU can be used to learn temporal dependencies (Gupta et al., 2023). As a result, models may become more robust, precise, and efficient as they can recognize and utilize a broader range of intricate patterns seen in the data (Gupta et al., 2023).

In line with recent advances in deep learning, we propose a reliable new hybrid model that combines 2DCNN and GRU. Our model takes advantage of both architectures to achieve high accuracy and efficiency. The proposed model architecture consists of several layers of 2DCNN to extract features from the input data and then GRU to analyze the temporal dependencies between the extracted features. It allows our model to capture spatial and temporal dependencies in the data, critical for many tasks such as image and time series analysis. Our main goal is to offer an efficient and reliable model that can be used in various applications and tasks.

**FIGURE 2:**
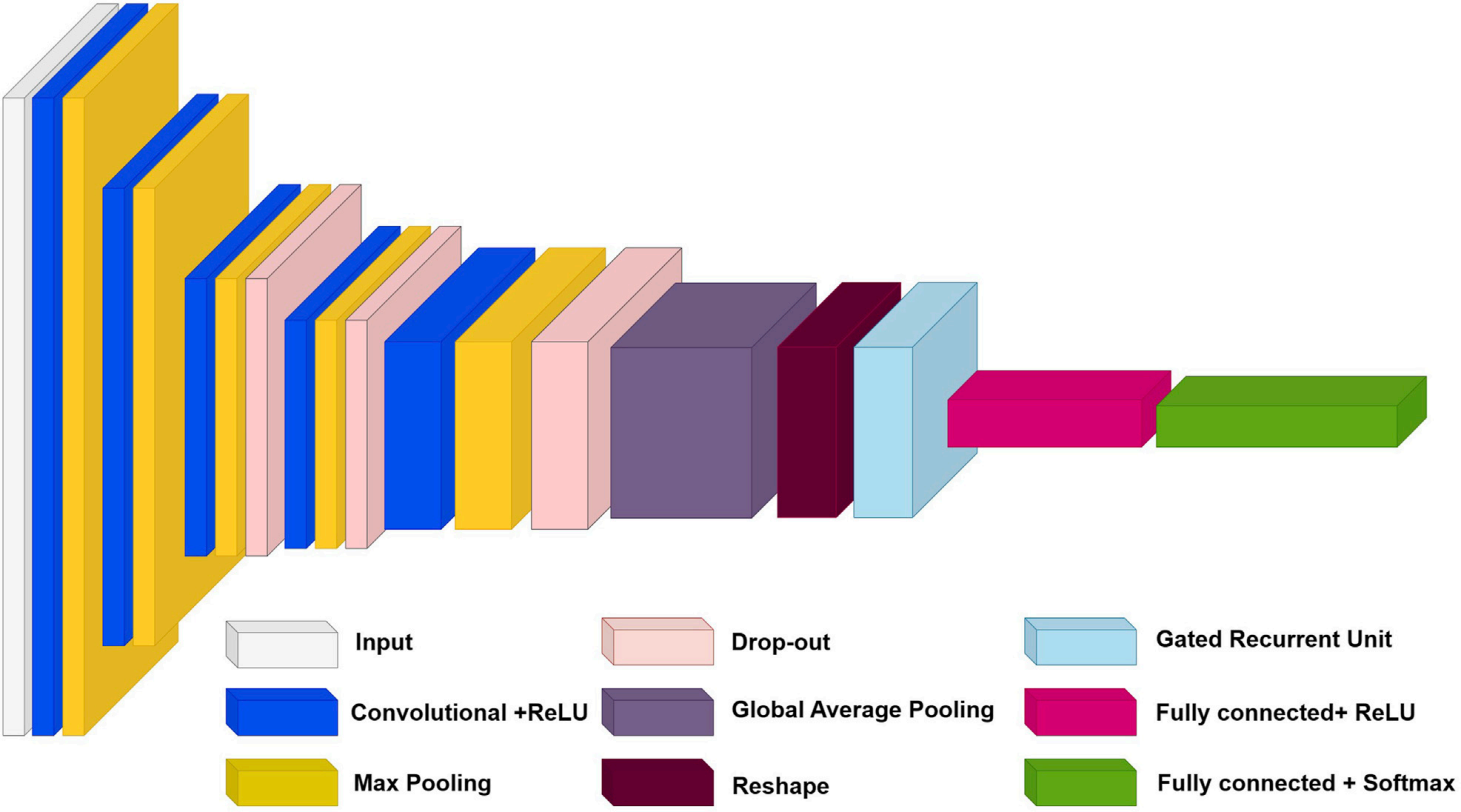
The architecture of the proposed hybrid model combining 2DCNN and GRU.

In this section, we will also take a closer look at the architecture of our model and discuss each of its layers. We will also present a table with model parameters and a description of each layer. The architecture of our model is shown in Figure 2. This figure shows the structure of our model, including all the layers and their order within the architecture. The roles of each layer are described as follows.

1. The input layer (Silver module in Figure 2): In our architecture, the input layer accepts 227 × 227 images with three color channels. This data is then sent over the network for further processing.
2. Five 2DCNN layers with ReLU activation (Blue modules in Figure 2): These layers are used to extract features from the input data. Each layer consists of several convolutional filters that sweep over the input data and transform it into feature maps. A ReLU activation function is applied to the output of each convolutional layer to add nonlinearity.
3. Five max pooling layers (Yellow modules in Figure 2): These layers reduce the dimensionality of feature maps while preserving the most essential features. It helps reduce the number of model parameters and increases its invariance to small changes in the input data.
4. Three dropout layers (Bronze modules in Figure 2): These layers randomly turn off some neurons during training to prevent overtraining. It helps the model generalize better to new data.
5. One Global Averaging Layer (Grey module in Figure 2): This layer averages information across the entire spatial dimension of each feature map while preserving depth. It allows the model to focus on global features.
6. Reshape Layer (Burgundy module in Figure 2): This layer reshapes the input data to match the next GRU layer.
7. GRU Layer (Cyan module in Figure 2): This layer analyzes the temporal dependencies between the extracted features. It uses gate mechanisms to control the flow of information.
8. First fully connected layer (Pink module in Figure 2): This fully connected layer has a ReLU activation function. This layer performs classification based on the extracted features. It transforms high-level features into class predictions.
9. Second fully connected layer (Green module in Figure 2): This is a fully connected layer with a SoftMax activation function. The SoftMax function converts the outputs of the last fully connected layer into class probabilities, ensuring that the sum of all probabilities equals one. It allows the outputs to be interpreted as membership probabilities in each class.

The model parameters and training hyperparameters, including the filters, activation functions, outputs, and types of each layer, are described in Supplementary Table S1. This table provides detailed information about each layer and helps the reader better understand the functioning of our model.

#### 2.3.4 Training details

The training hyperparameters are presented in Supplementary Table S1. The optimization method chosen was the Adam algorithm with a 0.001 learning rate and 1e-6 decay, while the loss function used was the categorical cross-entropy metric. Compared to alternative optimizers, the Adam method usually exhibits accelerated dynamics during the neural network training process. The model applies a batch size of 16 and limits the number of training epochs to 400. The proposed neural network is implemented using Python 3.10 and TensorFlow package 2.10 and the training process was performed on a computing platform with a 12th Gen Intel^®^ CoreTM i7-12700 2.10 GHz processor, 64-bit operating system, and 32GB of RAM.

## 3 Results

This section shows the outcomes of our experiments on our model for classifying four shockable arrhythmias types. It also compares these outcomes to previous studies and discusses their practical applications.

### 3.1 Analysis of the electrocardiogram represented by the time-frequency scalogram

**FIGURE 3:**
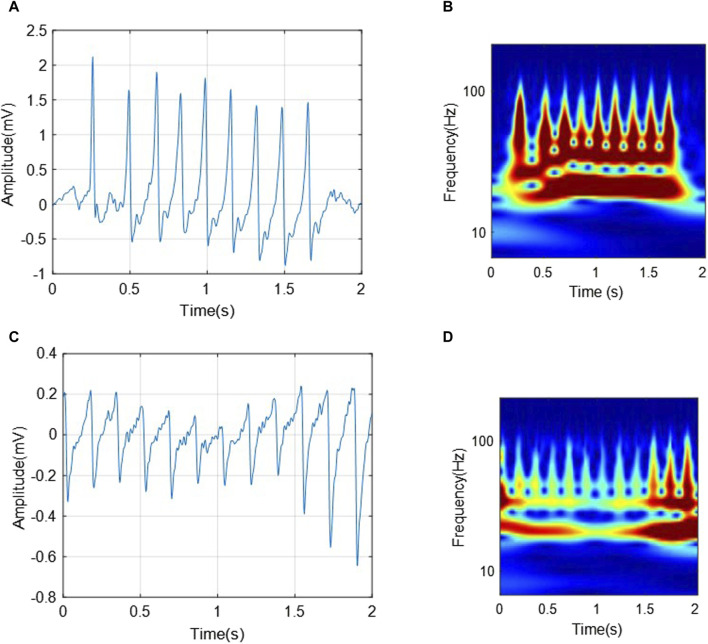
The transformations of two segments from C3 (VTHR) and C4 (VTTdP) classes, respectively. **(A)** A 2-s-ECG segment from C3; **(B)** The corresponding scalogram of C3 ECG segment; **(C)** A 2-s-ECG segment from C4; **(D)** The corresponding scalogram of C4 ECG segment.

In converting a one-dimensional ECG signal from the time domain to the time-frequency domain using wavelet transform, the ECG signal is converted into a two-dimensional matrix (Byeon et al., 2019). It allows multi-resolution signal analysis, enabling an in-depth analysis of its properties. Figure 3 shows examples of the obtained scalograms using CWT of the segmented ECG signals with a length of 2 seconds from the C3 and C4 classes, respectively. The difference between ECGs and scalograms in.

Figure 3 can be analyzed from two aspects as follows:

• The two ECG segments show characteristic wave changes and interval associated with corresponding arrhythmias, respectively. For example, VTHR (C3) may present as rapid and regular ventricular QRS complexes, while VTTdP (C4) may present as a rapid and pulsatile change in QRS amplitude around the isoelectric line.
• The VTHR (C3) scalogram shows the high frequency and regular components associated with this arrhythmia. In contrast, the VTTdP (C4) scalogram shows rotational signal amplitude changes around a specific frequency, which is characteristic of this tachycardia.

Thus, it can be seen that the transformation of the ECG signal associated with arrhythmias from the temporary domain into a time-frequency one using CWT can provide a complete and more accurate signal characteristic. It can help in the classification of various arrhythmias, as well as in the development of new diagnostic and monitoring strategies for heart diseases.

### 3.2 Model performances

#### 3.2.1 Experimental validation results

In this research work, the original data set was divided into three distinct samples: training, validation, and testing to ensure thorough evaluation and development of the model. The rationale for this strategy decision was to achieve balance across many aspects of the deep learning process, including training, hyperparameter tuning, and the ultimate assessment of model performance. Prior to partitioning the data, all images are normalized to values within the range of [0 255]. The images were normalized by dividing each pixel value by 255. Consequently, all the pixels in the image are adjusted so that their values are confined inside this specific range. This crucial preprocessing step enhances model performance by ensuring all input variables are normalized to the same scale.

The training set, containing 80% (3200 fragments) of the original data, served as the primary data set for training the model. This amount of data was chosen based on the model’s desire to learn patterns and generalizations from many examples, allowing it to better learn from various scenarios. A validation set of 10% (400 fragments) of the original data was used to tune the model’s hyperparameters and monitor its performance. This sampling allowed for the necessary iterations of model tuning to achieve optimal results and prevent overfitting. The test set also comprised 10% (400 fragments) of the original data and was used to ultimately evaluate the performance of the trained model. It remained “hidden” from the model during training and tuning, ensuring an objective measurement of its ability to generalize knowledge to new data. This strategy of splitting training, validation, and testing sets provided a framework and methodology for developing, evaluating, and tuning the model while considering the need to train on a large amount of data, test its performance, and avoid overfitting. Table 2 shows the performances of our proposed method validated by the test dataset. The average classification accuracy for all four classes is 97.75%. It testifies to the model’s ability to identify and distinguish each class’s features effectively. The average classification precision, specificity, recall (sensitivity), and F1-score for all four classes are 97.75%, 99.25%, 97.75%, and 97.75%, respectively. It is noticeable that the model showed promising results in these measures for all classes, which indicates a balance in its ability to classify both positive and negative examples correctly.

**TABLE 2:** The performance of our proposed method for all classes.

Type of arrhythmias	Precision (%)	Specificity (%)	Recall (%)	F-score (%)
**C1**	100	100	93	96
**C2**	0.97	99	100	99
**C3**	99	99.7	98	98
**C4**	95	98.3	100	98
**Average**	97.75	99.25	97.75	97.75

**FIGURE 4:**
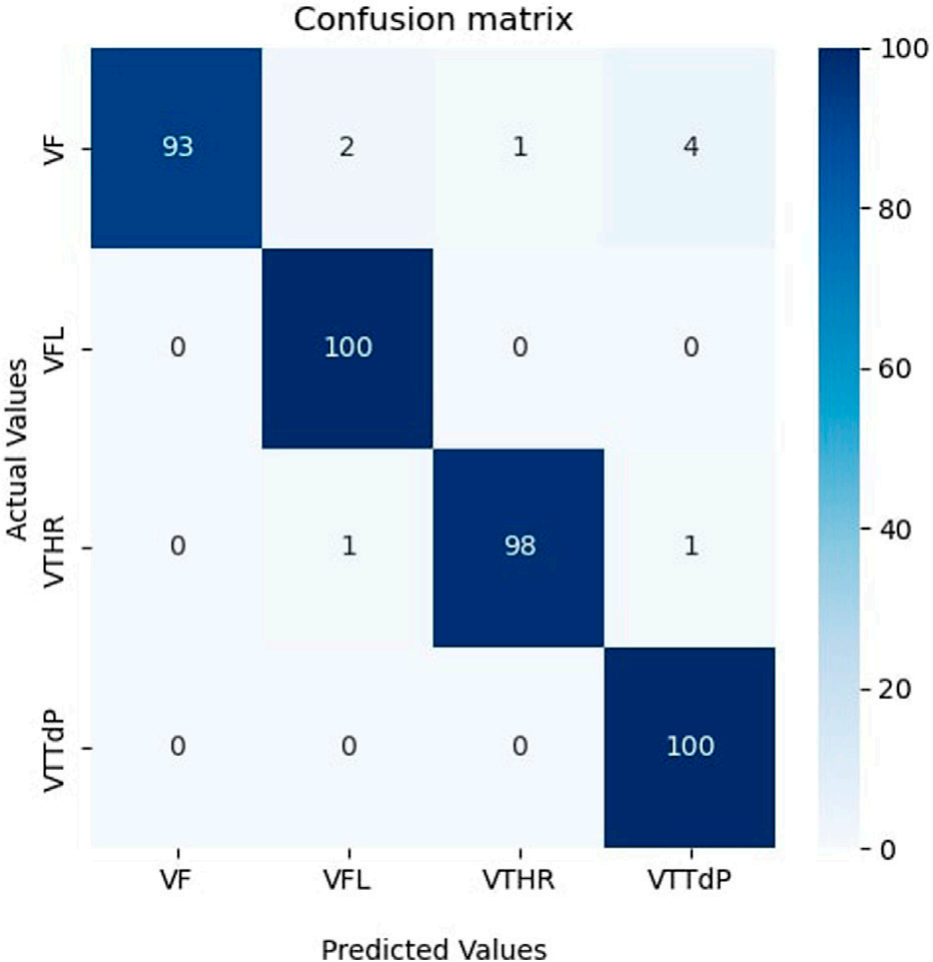
Confusion matrix on the unseen testing set.

The results obtained were impressive. The model achieved high classification results on the test samples, which confirms the effectiveness of the proposed hybrid model with the combination of CWT. It indicates that CWT could highlight vital temporal features in the data, and the hybrid model successfully used these features to make accurate classifications. Achieving high classification results in this problem is of great practical importance. Furthermore, an analysis of the confusion matrix in Figure 4 revealed that the model made the most errors when classifying Ventricular fibrillation (VF) and high-rate ventricular tachycardia (VTHR) classes. However, even in these cases, the model showed an acceptable ability and accuracy to separate classes. The confusion matrix of Figure 4 shows that most of the samples were correctly classified. A small number of incorrectly classified samples suggests that our model has accurately learned the features and data patterns. Nevertheless, we note that in two classes several samples are incorrectly classified, which indicates that our model is not ideal and may have some restrictions. Further research will be carried out to determine specific areas where the model requires improvement. The results show that our model has potential for several uses, including disease classification and medical diagnostics. Classifying medical images according to their content is one of the possible applications of our methodology.

**FIGURE 5:**
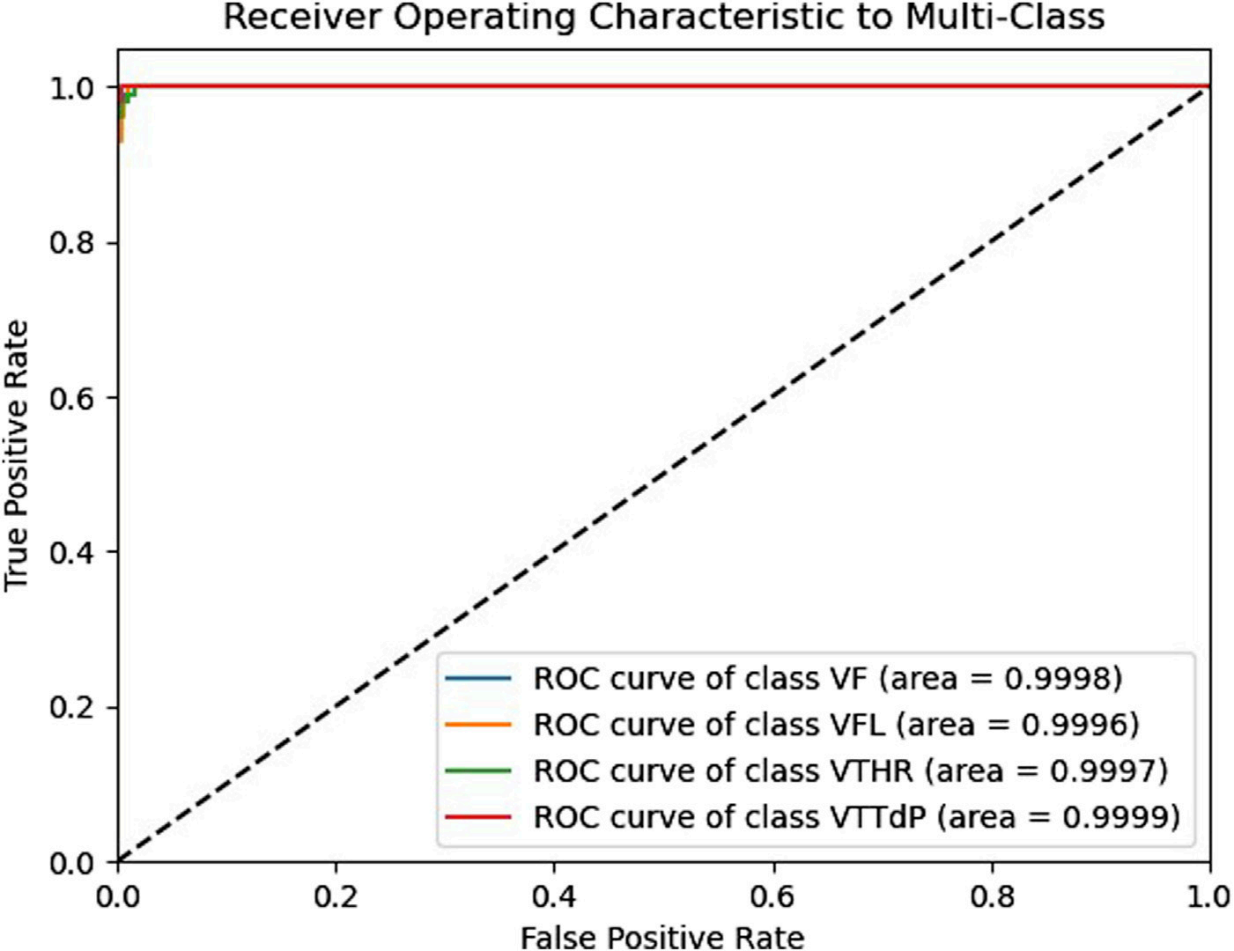
The Receiver operating characteristic of the proposed model.

The receiver operating characteristic (ROC) curve is crucial for assessing a classifier’s effectiveness. It displays the correlation between the true positive rate (TPR) and the false positive rate (FPR) at various thresholds. A random classifier’s area under the curve (AUC) is 0.5, whereas an ideal classifier’s area under the curve (AUC) is 1. According to Figure 5, our model shows excellent AUC results for all four classes, which are close to 1. The ROC curves are close to the top left corner of the graph, indicating high TPR and low FPR for all classes. It demonstrates that our model effectively distinguishes between different classes and can be used for robust classification in real-world applications such as the classification of medical imaging, etc., where accurate data classification is critical. These results confirm the superiority of our model and its efficient classification ability.

**FIGURE 6:**
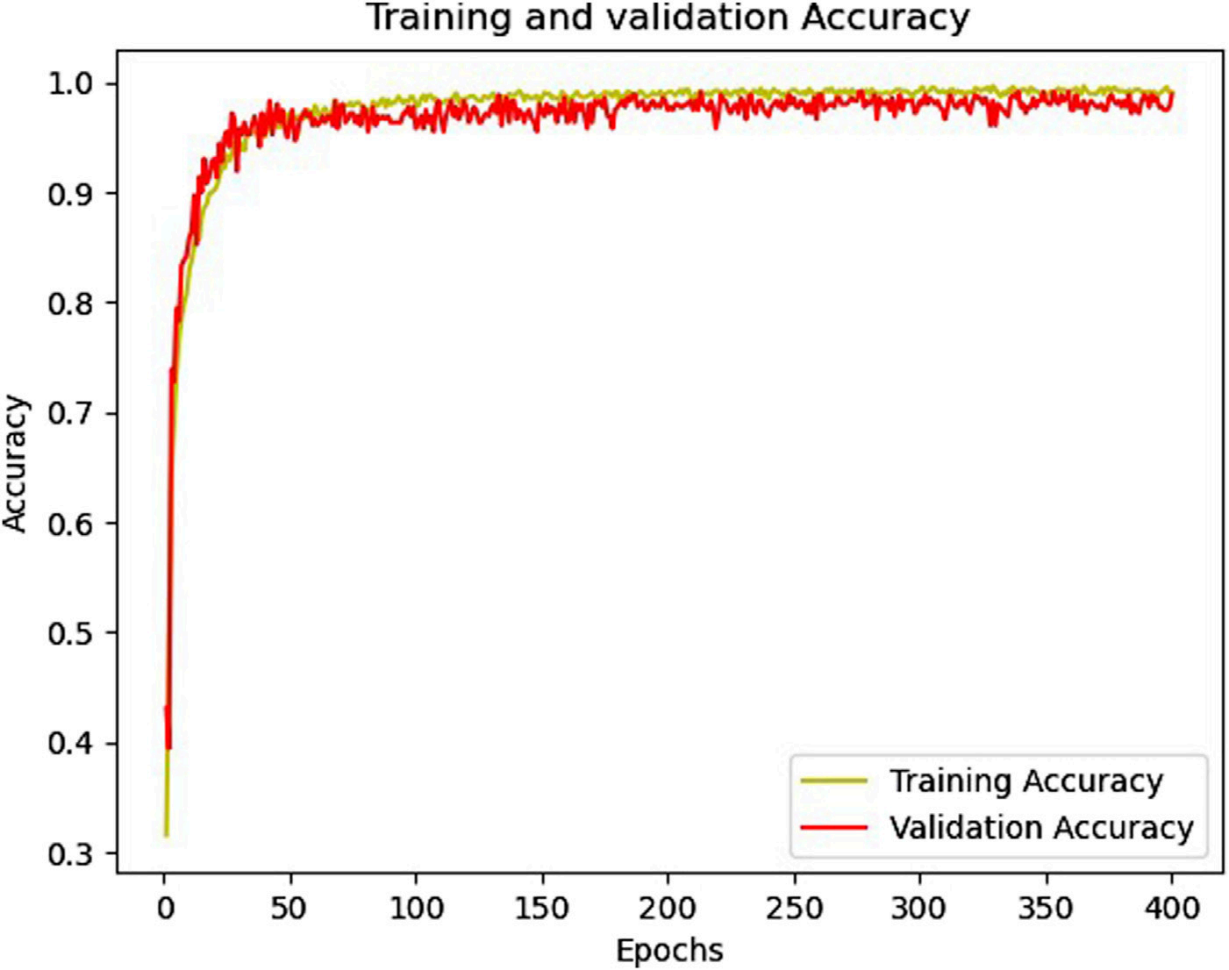
The training and validation accuracy curves.

**FIGURE 7:**
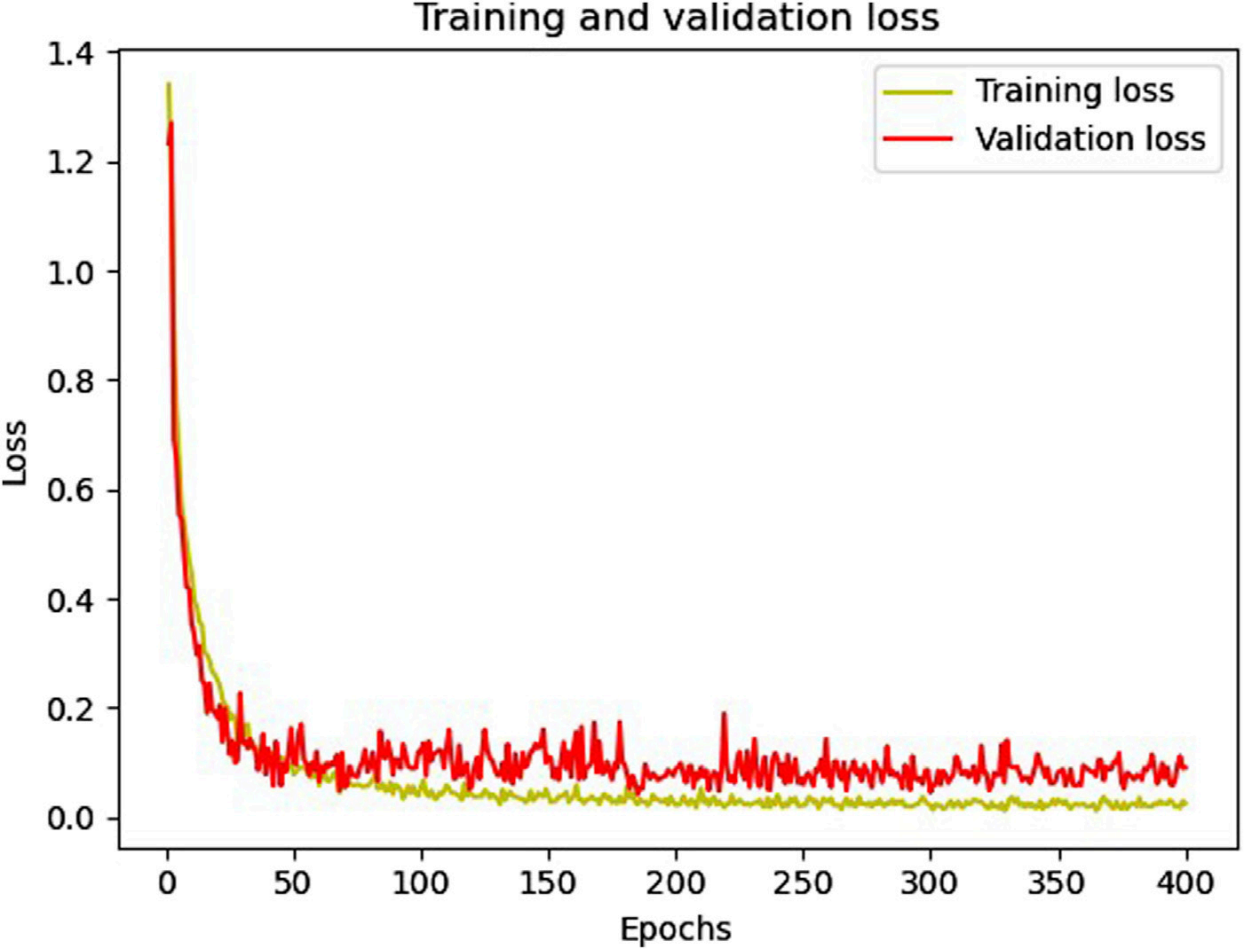
The training and validation loss curves.

Figures 6, 7 show the accuracy and loss function curves acquired during the training of the proposed model for 400 epochs, respectively. Upon examining the accuracy curve, it is evident that after 140 epochs, both training and validation accuracy settle at above 98%, signifying highly efficient classification on the considered database. The cross-entropy function performs well, as evidenced by the loss plots staying comparatively steady during the training phase and the loss function remaining steady between 0 and 0.2.

#### 3.2.2 Cross-validation results

Cross-validation is a crucial technique for evaluating model performance and choosing the best hyperparameters. Using this technique, we can assess how well our model will handle fresh data that it has never seen before. We employed five cross-validations during model training to guarantee its reliability.

The procedure involved dividing our data into five subsets. The model was then trained on four, leaving one as a test set. This process was repeated several times, with each repetition a different subset serving as the test set. This approach provided a more reliable assessment of model performance.

We use Cross-validation to ensure our model can generalize information from the data without overfitting. It is essential to guarantee that our proposed model can make correct predictions based on fresh data that it may come across in practical applications. Table 3 presents the accuracy and loss results of the five cross-validations. It can be seen from Table 3 that all accuracy values exceed 96%, which indicates the high efficiency of the developed model. These high scores indicate the model’s ability to generalize successfully to new data, which is an essential factor in the context of its potential application.

**TABLE 3:** The results of five cross-validation.

Fold No	Accuracy	Loss
1	0.969	0.155
2	0.969	0.111
3	0.96	0.219
4	0.975	0.131
5	0.961	0.172
**Mean**	0.967	0.158

## 4 Discussions

### 4.1 Comparison with other state-of-the-art models

This section presents a comparative study between our proposed model and six state-of-the-art (SOTA) deep learning models for ECG analysis and classification on the same database. Several factors are compared: accuracy, sensitivity, specificity, precision, F1 score, model parameters, and training time per epoch. Table 4 shows the comparison results for the ECG classification task.

**TABLE 4:** Comparison of the proposed model with SOTA models for the four arrhythmias classification.

Model	Accuracy	Sensitivity (recall)	Specificity	Precision	F1 score	Model parameters	Training time per epoch
**VGG19**	0.945	0.945	0.981	0.948	0.946	26.4 million	190 s
**Xception**	0.945	0.945	0.98	0.945	0.945	46.5 million	80 s
**InceptionV3**	0.91	0.91	0.97	0.912	0.91	34.9 million	145 s
**GoogLeNet**	0.935	0.936	0.978	0.935	0.935	4 million	14 min
**MobileNETV3**	0.923	0.923	0.974	0.924	0.923	3 million	14 s
**VGG16**	0.967	0.967	0.989	0.967	0.929	21 million	172 s
**Our Model**	**0.9775**	**0.9775**	**0.9925**	**0.9775**	**0.9775**	**208 thousand**	**26 s**

Bold values refer to the optimal results.

**FIGURE 8:**
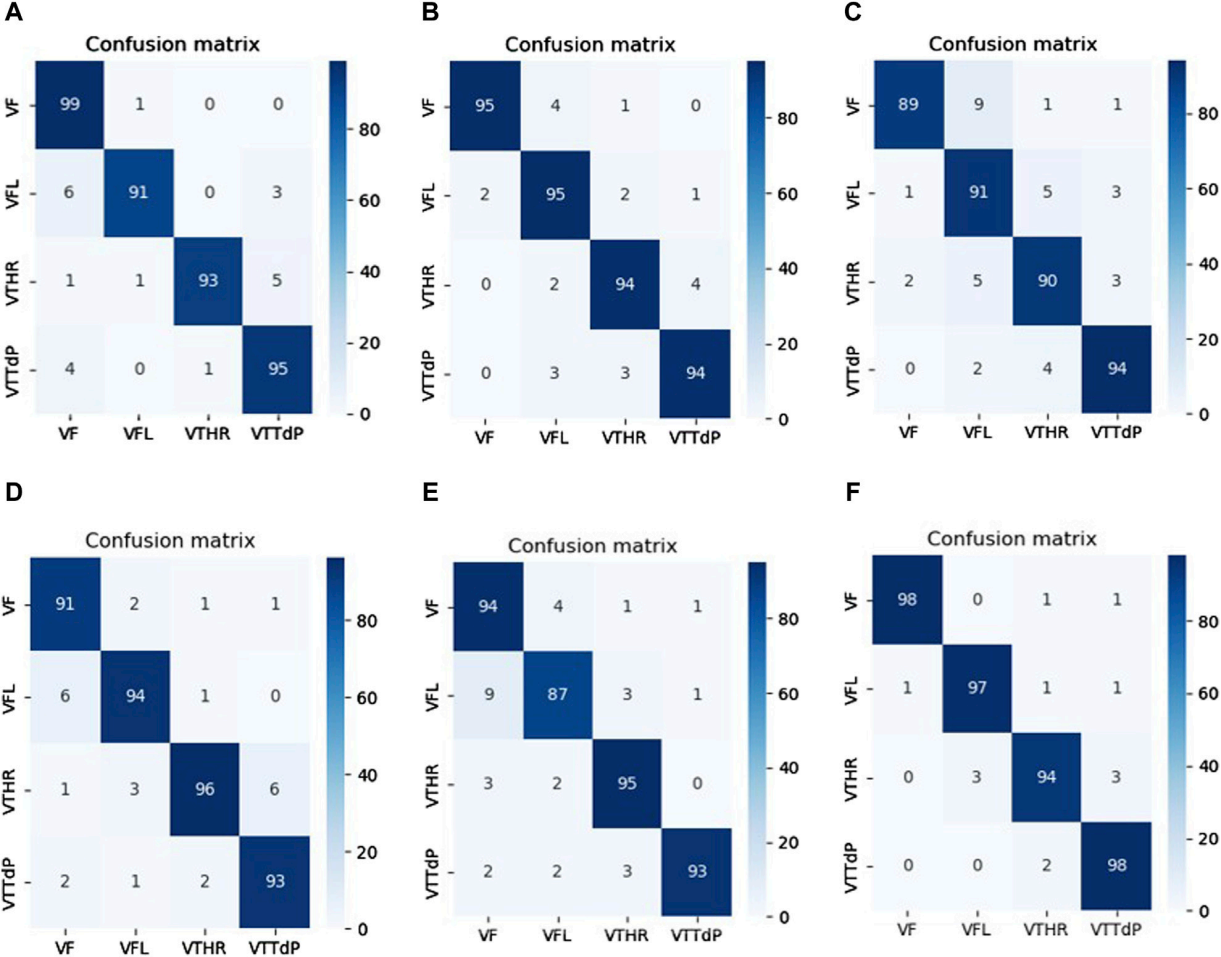
Confusion matrices of the SOTA deep learning models. **(A)** VGG 19. **(B)** Xception. **(C)** InceptionV3. **(D)** GoogLeNet. **(E)** MobileNETV3. **(F)** VGG16.

Table 5 proves that our developed model is superior to other deep models, which demonstrates its efficiency and reliability. We believe that our model is a promising solution for the four arrhythmias classification using deep learning. Figure 8 shows the confusion matrices of the six other deep models.

**TABLE 5:** Assessment of performance indicators for all approaches.

Model type	Precision (%)	Specificity (%)	Recall (%)	F-score (%)
2DCNN alone	0.97	0.99	0.97	0.97
GRU alone	0.909	0.9683	0.905	0.905
**Our model(2DCNN + GRU)**	**0.9775**	**0.9925**	**0.9775**	**0.9775**

Bold values refer to the optimal results.

**FIGURE 9:**
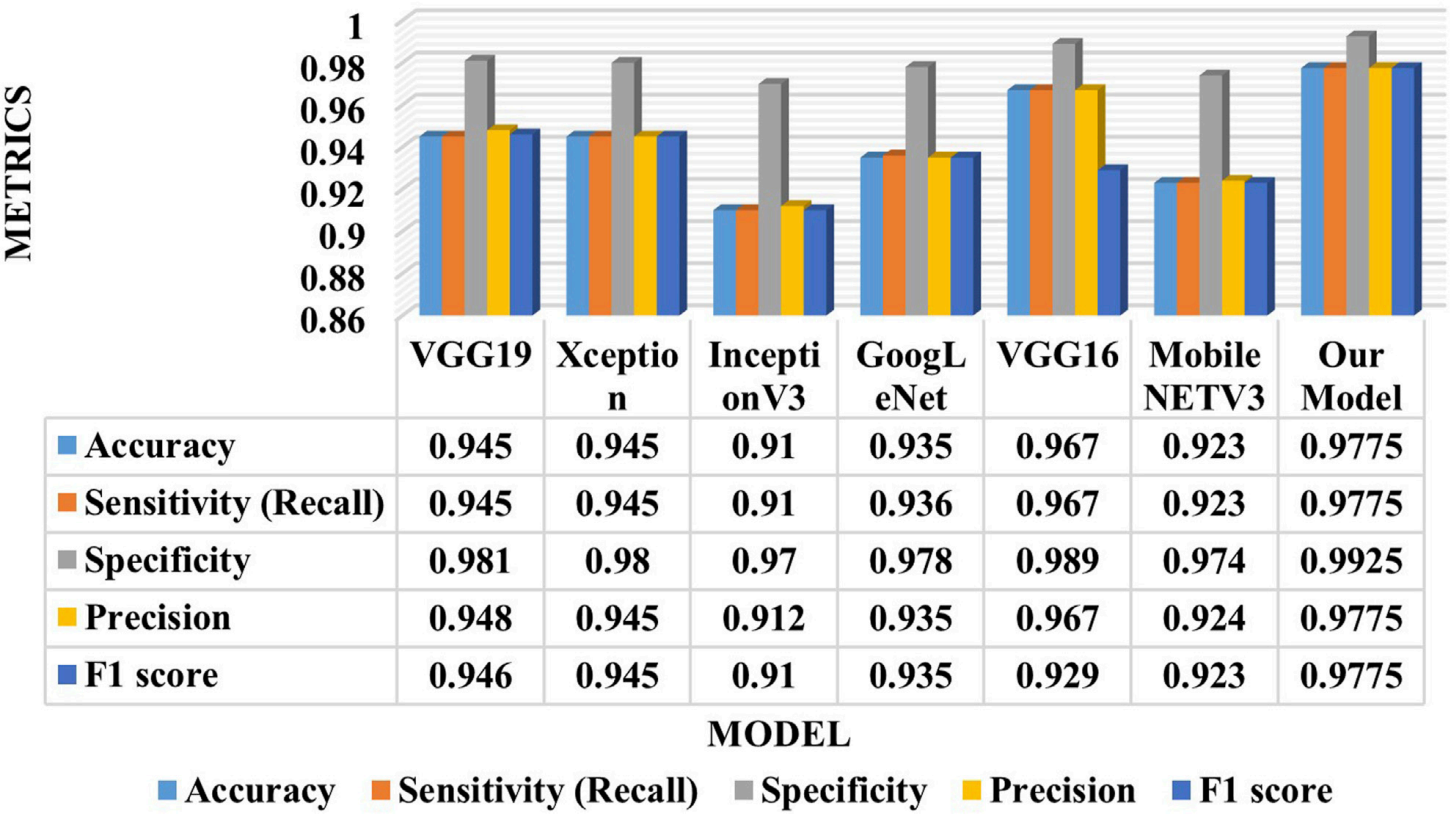
Graph comparison of models’ performance for the four arrhythmias classification.

Compared between the confusion matrix of our proposed model shown in Figure 4 with the confusion matrices of SOTA shown in Figure 8, our model demonstrated outstanding and accurate classification with only a small number of errors. It emphasizes its reliability and high level of accuracy in comparison with the other six models. Figure 9 displays a visual graph comparison of the performance metrics of our model and six different SOTA deep learning models on the same database. By analyzing this graph, we can highlight key metrics and compare the performance indicators of each model.

### 4.2 Ablation experiment results

To verify and compare the performance of our approach, a comparison is made between the hybrid and individual models. This comparison is based on a thorough performance analysis, which is presented in Table 5.

The results in Table 5 clearly show that the hybrid model works better than a stand-alone approach. The observed performance gains validate the benefits of hybrid techniques in optimizing model analysis performance and highlighting their high potential.

### 4.3 Generalization ability study

To evaluate the generalization ability of our model on other databases, we resorted to using the MIT-BIH database (Moody and Mark, 2001), which is firmly rooted in the field of ECG analysis and research and provides a wealth of data for our research purposes. (This database is available on this website: https://physionet.org/content/mitdb/1.0.0/). From this database, we selected four balanced classes (Atrial premature beat (A), Left bundle branch block (L), Normal (N), and Right bundle branch block (R)). The annotations were used to segment the signals of these classes, so each segment lasted 0.6 s. After that, all segments were transformed into scalograms as described in Section 2.2. It is worth noting that the data of these classes are actual, i.e., synthesized data was not generated. Impressively, the results outperformed the database under consideration and validated our model’s exceptional performance on actual data. The results of the proposed model on this database are presented in Table 6.

**TABLE 6:** The performance of our proposed method on the MIT-BIH database.

Type of arrhythmias	Precision (%)	Specificity (%)	Recall (%)	F-score (%)
A	95	98.3	97	96
L	100	100	100	100
N	98	99.3	95	96
R	99	99.7	100	100
Average	98	99.33	98	98

**FIGURE 10:**
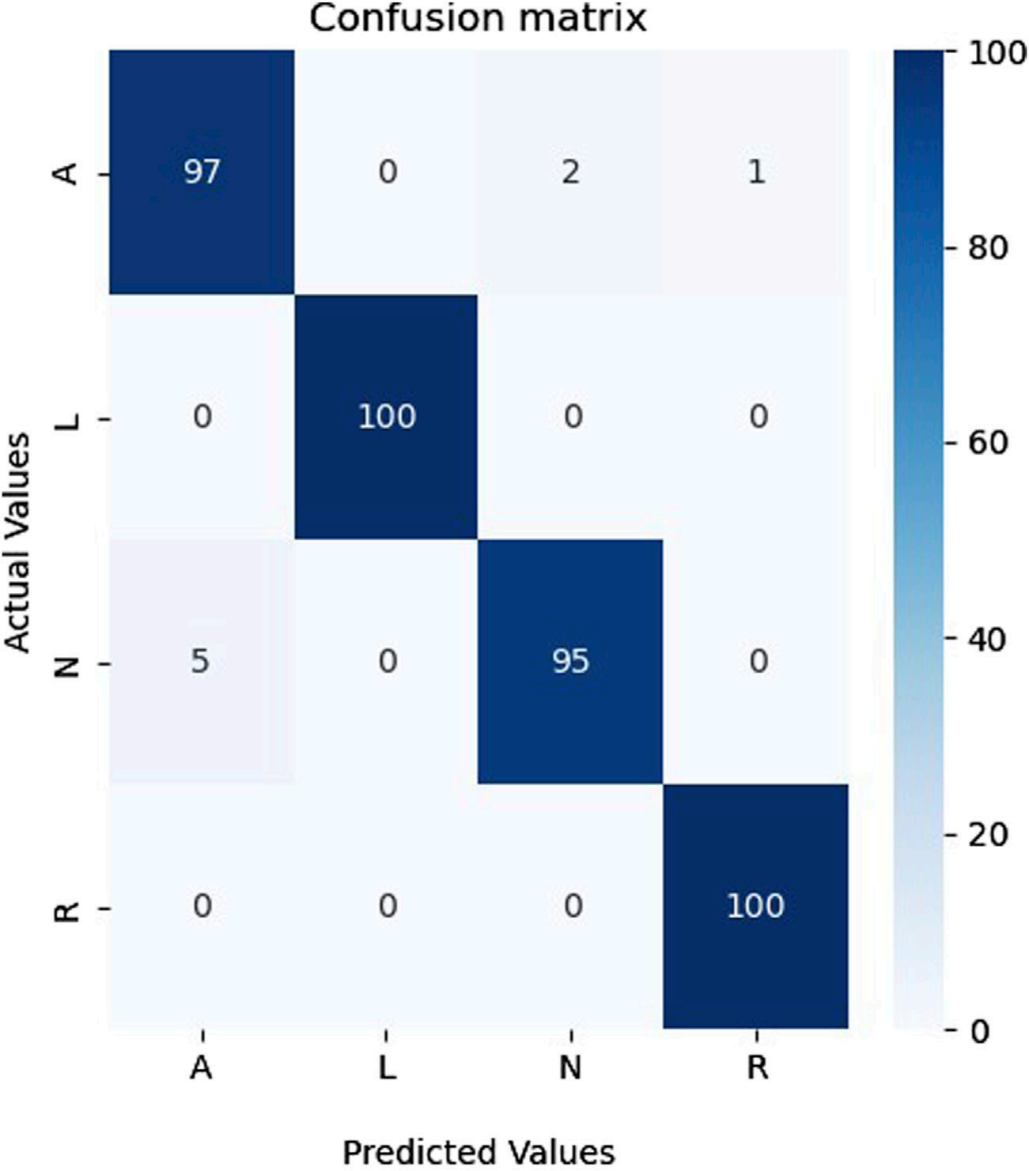
The confusion matrix for the four classes from the MIT-BIH database.

The outcomes in Table 6 show that our proposed model can successfully adapt to new data, which is well supported by the results achieved. It is crucial to emphasize that our model’s adaptability may be shown in a range of datasets and not just in the database used for building the model, demonstrating its good generalization ability and wide range of applications. Figure 10 shows the confusion matrix of the proposed model for classifying A, L, N, and R classes from the MIT-BIH database.

As shown in Figure 10, the proposed model could recognize all four classes and made only eight errors, proving the performance and generalization of the proposed model on a different database that contains only actual data.

### 4.4 Model applicability validation

We utilized the Brain Tumor dataset (Bhuvaji et al., 2020) to validate the applicability of our proposed model on additional image datasets. This dataset comprises brain magnetic resonance imaging (MRI) images categorized into four groups: no tumor, pituitary tumor, malignant tumor, and benign tumor. To aid researchers in developing and evaluating machine learning algorithms for detecting and categorizing brain tumors, the 3,064 image collection is split into training and testing sets. The data sources consist of 512 × 512-pixel resolution MRI images gathered from multiple sources and displayed in PNG format. The Kaggle platform offers a database for downloading (Bhuvaji et al., 2020). A benign tumor (PT), malignant tumor (MT), and pituitary tumor (PT) were the three categories chosen to guarantee class balance. Normalization and image resizing to match the model’s input size were among the standard pre-processing operations carried out on the data. Table 7 presents the results of the classification.

**TABLE 7:** The performance of our proposed method on the brain tumor database.

Type of arrhythmias	Precision (%)	Specificity (%)	Recall (%)	F-score (%)
**GT**	96	98.4	87	91
**MT**	88	93	95	91
**PT**	98	98.9	100	99
**Average**	94	96.76	94	93.66

**FIGURE 11:**
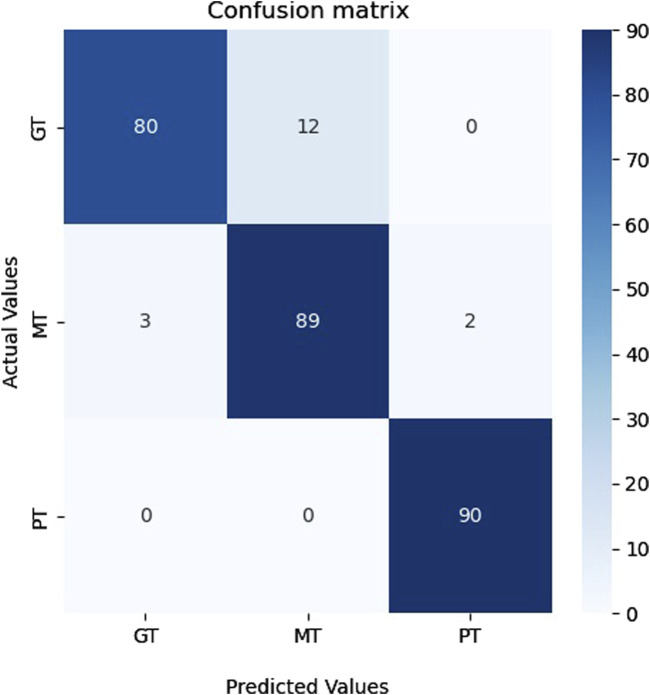
The confusion matrix for the Brain Tumor classes.

Table 7 shows that our model attained a specificity of over 96%, indicating that the proposed model can be adapted and applied to other image datasets. Figure 11 shows the confusion matrix of classification results. As can be seen in Figure 11, the proposed model successfully recognized the majority of representatives of each class and made only 17 errors, confirming this model’s effectiveness and applicability to other image data sets.

### 4.5 Comparison with related works

To further verify the cruciality of the results achieved, Table 8 compares the results of our proposed model with previous studies in the main performance indexes for classification. It is worth noting that the results achieved exceed those of the best similar studies. It further confirms the effectiveness of the original proposal of the model architecture and approach to solving the classification problem for identifying VFL (C1), VF(C2), VTTdP(C3), and VTHR (C4).

**TABLE 8:** Comparisons with previous related studies in main performance indexes.

Author	Approach	Length of ECG segment: (s)	Main performance index
Rajendra Acharya et al. (2018)	1D-CNN	2	Accuracy:93.18%, Sensitivity:95.32%, Specificity: 91.04%
Ba Mahel and Kalinichenko, (2022)	The ECG segments are represented as 100 × 100 images and 2-dimensional convolutional neural network (2DCNN) was used for the classification	0.4	Accuracy: 68.6% Recall (Sensitivity): 85.4% Precision: 63.9% F1: 73.1%
Tripathy et al. 2(2016)	Random forest Classifier	8	Accuracy: 94.07% Sensitivity:94.37% Specificity: 94.73%
Alonso-Atienza et al. (2014)	Support vector machine classifier	8	Sensitivity: 92% Specificity: 97%
Liu et al. (2023)	Channel-based attention and bidirectional LSTM	0.9	Precision: 91.1%, F1:90.8%
Yang et al. (2021)	Cascaded convolutional neural network	9 to 91	F1 score: 86.5%
Giriprasad Gaddam et al. (2021)	CWT + CNN	1.4	Accuracy: 95.31% Sensitivity: 94.21% Specificity: 93.26% Precision: 93.12%
Jeong and Lim, (2021)	2D CNN	2 to 3	F1: 78%
Zhang et al. (2022)	3D recurrence plot analysis and deep learning	5	F1: 92.5%
Lee et al. (2021)	One-dimensional morphological Features with XGBoost machine learning algorithm	10	Accuracy:90.46%, Sensitivity:89.2%, Precision:90%, F1:89.5%
**Our proposed method**	CWT approach and hybrid Deep learning model 2dCNN + GRU	2	Accuracy: 97.75% Recall (Sensitivity): 97.75% Specificity: 99.25% Precision: 97.75% F1: 97.75%

### 4.6 Model hyperparameters tuning

Optimizing parameters such as the learning rate and batch size is an essential stage in the training procedure of neural networks. Correctly selecting model hyperparameters is crucial for both the quality and efficiency of model training. The batch size defines the balance between the frequency of gradient updates and the algorithm convergence rate. Hence, it is imperative to strike a perfect balance between precision and efficiency to train neural networks, guaranteeing optimal outcomes effectively. Table 9 displays various values employed for model tuning and the highest accuracy attained.

**TABLE 9:** The performance of our proposed method using different hyperparameters.

Learning rate	Decay	Batch size	Optimizer	Accuracy
0.001	1e-6	16	Adam	0.9775
0.001	1e-6	32	Adam	0.972
0.001	1e-6	64	Adam	0.967
**0.001**	**1e-6**	**128**	**Adam**	**0.9775**
0.0001	1e-6	128	Adam	0.91
**0.001**	**1e-6**	**256**	**Adam**	**0.9775**

Bold values refer to the optimal results.

It can be seen from Table 9 that the best performance was achieved using the Adam optimizer with a learning rate of 0.01, 1e-6 decay, and a batch size of 16, 128, and 256, respectively. This parameter choice highlights the importance of adequately tuning hyperparameters to achieve maximum model performance.

### 4.7 Analysis of the model performance on imbalanced original data

To further validate the performance of our model, we experimented it on the actual imbalanced data without oversampling using the SMOTE method. The total number of actual samples without using the SMOTE method is 587. From the analysis of the data presented in Table 1, it is evident that the classes exhibit a significant imbalance, which prevents practical training of the model. This experiment was based on the imbalanced original data to validate the model efficiency. The evaluation metrics of this experiment are presented in Supplementary Table S2, while the confusion matrix is shown in Supplementary Figure S1. Despite the big class imbalance, the model achieved a precision of 82.75%, a specificity of 94.58%, a recall of 82%, and an F1-score of 82%. These high average values further confirm the model’s high efficiency.

### 4.8 Explanation of model predictions

This section presents the results of using the LIME (Local Interpretable Model-agnostic Explanations) method to explain the model’s predictions (Tulio Ribeiro et al., 2016). The LIME method was applied to identify the most significant regions in the input data contributing to the model’s decision-making.

To explain the model’s predictions, we used LIME, which allows us to identify the most critical superpixels (segments) in the image that influence the prediction result. Scalograms with the size of 227 × 227 pixels with three color channels were used as input data. During the experiments, the LIME method showed high efficiency and information content. Visualization of the masks obtained using LIME, as shown in Supplementary Figure S2, allows us to identify the areas of the scalogram that impact the model’s prediction the most. The graphs show the original scalograms and their corresponding masks; the most significant areas are highlighted in yellow.

Results from the LIME method show that the model focuses on specific parts of the scalogram that are most relevant for the classification. These regions coincide with the important features of the input data of ECG scalograms, confirming that the model’s predictions are correct and interpretable.

Analysis of masks obtained using LIME allows us to better understand which parts of the scalogram are most important for the model. For example, in the presented mask in Supplementary Figure S2, it can be seen that the model pays special attention to areas located closer to the bottom and central part of the scalogram. It indicates that specific data frequency components and time segments are crucial to the model when making decisions.

Thus, the LIME method used to explain model predictions has shown promising results. Highlighting significant areas in scalograms allows us to understand the model’s decision-making process better and verify its operation’s correctness. This approach increases confidence in the model and identifies potential areas for improvement.

### 4.9 Comparison with traditional ECG classifiers

To further evaluate the performance of our proposed deep model, we also conducted a comparative analysis using traditional ECG classification methods such as k-nearest neighbours (kNN), support vector machine (SVM), decision trees (DT), random forests (RF), naive Bayes classifier (NB) and ensemble SVM method. The results of the comparative analysis with the related (Al-Shammary et al., 2024; Mondejar-Guerra et al., 2019; Pandey et al., 2020; Sharma et al., 2019; Wang et al., 2022) studies are presented in Supplementary Table S3. As we can see from the table, our deep model demonstrates superiority in all key metrics. Our model’s precision, recall, accuracy, and F1-score are significantly higher than traditional methods.

These results confirm that deep models have the potential for more accurate and robust ECG pattern recognition compared to traditional machine learning methods. The strong performance of our model is attributed to the deep neural network’s ability to effectively capture and process complex non-linear relationships in ECG data, resulting in improved overall classification accuracy.

### 4.10 Future work

The classification of the four high-risk arrhythmias is of sufficient significance because they represent the most dangerous and common heart rhythm disorders that can lead to death or disability. However, future research could more deeply explore the distinctions between low-risk, normal ECGs, and high-risk arrhythmias. Future research aims to broaden the application of the suggested approach by examining a more comprehensive range of arrhythmias encompassing high-risk and low-risk conditions, including normal ECGs. To maximize the accuracy and efficiency of the approach, more research is scheduled to be undertaken simultaneously. It involves thinking about incorporating other features and algorithms into the classification procedure.

In addition, to investigate the importance of differentiating high-risk arrhythmias from normal and low-risk arrhythmias, future studies will conduct a more in-depth analysis of the differences between these categories. It may include the use of additional parameters as well as the use of machine learning and deep learning techniques for more accurate differentiation. Also, within future research framework, a significant expansion of the classification area is planned, including up to 10 additional types of arrhythmias. This line of research aims to gain a deeper understanding of the diversity of cardiac arrhythmias and develop more universal methods for their detection. These improvements will allow a more detailed study of the characteristics of each type of arrhythmia, increasing the accuracy of the diagnosis. In addition, the methods presented in these studies (Sharma and Ramesh, 2018; Rahul et al., 2021; Chaitanya and Sharma, 2024) will be considered in future works further to improve the results and the accuracy of the models.

## 5 Conclusion

In this article, an approach based on a combination of continuous wavelet transform (CWT) and a novel hybrid neural network for solving the problem of four dangerous arrhythmias classifications was proposed in detail. The experiments and analysis of the results led to several important conclusions about the applicability of the proposed approach and its significance in daily real-time monitoring and clinical diagnosis.

The results demonstrate that combining CWT and the novel hybrid model leads to high accuracy in identifying dangerous arrhythmias. CWT allows us to extract critical time-frequency characteristics from the ECG data in the time domain, and the proposed model successfully captures these characteristics and uses them for accurate classification. This interplay between data analysis and deep learning techniques highlights their complementary nature. We achieved high accuracy, sensitivity, specificity, precision, and F1 - score for all four classes (97.75%,97.75%,99.25%,97.75%, and 97.75%, respectively). The reported outcomes significantly outperform the best results obtained by other studies using the same types of ECG data. However, it should be noted that further research is also of great importance. The possibilities of optimizing the neural network architecture, adapting the method to other data types and classes, and comparing with alternative approaches can expand our understanding of the domain and improve results.

Overall, this article highlights the importance of integrating data analysis and deep learning methods to solve complex classification problems successfully. The presented approach has the potential for further research and practical application, contributing to improving the quality of dangerous arrhythmia classification. Thus, the main advantages of our approach include, but are not limited to:

1) Improved model performance: Using synthetic data generated by the SMOTE method resulted in significant improvements in the performance of our deep learning models. It allowed for more accurate identification and classification of shockable arrhythmias based on ECG signals.
2) Solving the problem of class imbalance: The SMOTE method allowed us to effectively deal with the issue of class imbalance, which is especially important in medical classification problems, where some classes of arrhythmias may be rare. Balanced data promotes fairer and more accurate classifications.
3) Increased generalization ability: Using synthetic data helped models better generalize knowledge from the training set to new, real-world data. It improves the models’ ability to recognize arrhythmias in actual clinical situations.
4) Expansion of applicability: Our approach using synthetic SMOTE data can be successfully applied to other time series and signal-based classification problems, making it a universal method for solving problems in medical diagnostics and beyond.
5) Minimize data collection costs: Generating synthetic data allows us to increase the amount of training data without the need for costly collection of additional clinical data.

Our method utilizing synthetic SMOTE data offers several advantages by enhancing the precision and generalizability of deep learning models in arrhythmia classification tasks using ECG signals. This novel hybrid model can be successfully adapted and applied in various fields where accurate data classification is required, such as classification of medical imaging, etc. The results achieved can significantly impact improving the quality of clinical decision-making.

## Data Availability

Publicly available datasets were analyzed in this study. This data can be found here: https://physionet.org/content/ecg-fragment-high-risk-label/1.0.0/, https://physionet.org/content/mitdb/1.0.0/, https://www.kaggle.com/datasets/sartajbhuvaji/brain-tumor-classification-mri.
