# Supplementary material for: Advanced integration of 2DCNN-GRU model for accurate identification of shockable life-threatening cardiac arrhythmias: a deep learning approach
Source: Front Physiol. 2024 Jul 12;15:1429161. doi: 10.3389/fphys.2024.1429161 (PMC11272599; doi:10.3389/fphys.2024.1429161)
Supplement: Supplementary file 1 [file DataSheet1.pdf]

Supplementary Material

Advanced Integration of 2DCNN-GRU Model for Accurate Identification of Shockable Life-Threatening Cardiac Arrhythmias: A Deep Learning Approach

Abduljabbar S. Ba Mahel, Shenghong Cao, Kaixuan Zhang, Samia Allaoua Chelloug, Rana Alnashwan*, Mohammed Saleh Ali Muthanna

*** Correspondence:** Rana Alnashwan: roalnashwan@pnu.edu.sa

Supplementary Table S1. The parameters and hyperparameters of our proposed hybrid model

Layer	Filters /Neurons	Filter size	Stride	Padding	Size of the feature map	Activation function
**Input**	–	–	–	–	227 × 227 × 3	–
**Conv 1**	32	3 × 3	1	\`same\`	227 × 227 × 32	ReLU
**Max pool 1**	–	2 × 2	2	–	113 × 113 × 32	–
**Conv 2**	64	3 × 3	1	\`same\`	113 × 113 × 64	ReLU
**Max pool 2**	–	2 × 2	2	–	56 × 56 × 64	–
**Conv 3**	64	3 × 3	1	\`same\`	56 × 56 × 64	ReLU
**Max pool 3**	–	2 × 2	2	–	28 × 28 × 64	–
**Dropout 1**	Rate = 0.3	–	–	–	28 × 28 × 64	–
**Conv 4**	64	3 × 3	1	\`same\`	28 × 28 × 64	ReLU
**Max pool 4**	–	2 × 2	2	–	14 × 14 × 64	–
**Dropout 2**	Rate = 0.3	–	–	–	14 × 14 × 64	–
**Conv 5**	64	3 × 3	1	\`same\`	14 × 14 × 64	ReLU
**Max pool 5**	–	2 × 2	2	–	7 × 7 × 64	–
**Dropout 3**	Rate = 0.3	–	–	–	7 × 7 × 64	–

**Global Average Pooling**	–	–	–	–	64	–
**Reshape**	–	–	–	–	1 × 64	–
**Gated Recurrent Unit (GRU)**	128	–	–	–	128	activation="tanh",<br>recurrent_activation="sigmoid"
**Fully Connected 1**	–	–	–	–	32	ReLU
**Fully Connected 2**	–	–	–	–	4	Softmax
**Training Hyperparameters and Specifications**						**Trainable parameters**
**Optimizer**	**Learning rate**	**Decay**	**Batch size**	**Epoch**	**Loss function**	208k
Adam	0.001	1e-6	16	400	Categorical cross entropy	

Supplementary Table S2. Model performance on imbalanced real data

Type of arrhythmias	Precision (%)	Specificity (%)	Recall (%)	F-Score (%)
**VFL(C1)**	89	98	80	84
**VF(C2)**	91	94	83	87
**VTTdP(C3)**	71	96	71	71
**VTHR(C4)**	80	90	94	86
**Average**	82.75	94.58	82	82

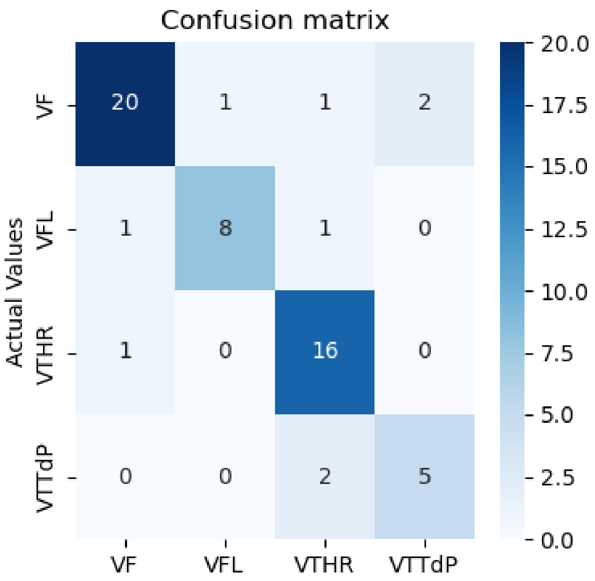

Supplementary Figure S1 | Confusion matrix of the model performance on imbalanced original data.

Supplementary Table S3. Comparison of performance of different ECG classification methods

Study	Classifier/Method	Metrics, %
**Pandey [60]**	RF<br>KNN<br>SVM<br>Ensemble SVM	Acc = 93.45<br>Acc = 72.56<br>Acc = 90.09<br>Acc = 94.40
**Sharma [61]**	KNN	Acc = 94.5
**Mondejar-Guerra [62]**	Ensemble SVM	Acc = 94.50
**Wang [63]**	EasyEnsemble	Acc= 95.6
**Al-Shammary [64]**	KNN<br>RF<br>SVM<br>NB<br>DT<br>Chi-square	Acc = 95.6, F1-score = 83.36, Pre = 83.48, Rec = 84<br>Acc = 77, F1-score = 73.20, Pre = 76.18, Rec = 77<br>Acc = 78, F1-score = 72.34, Pre = 83.14, Rec = 78<br>Acc = 74, F1-score = 74.49, Pre = 75.22, Rec = 74<br>Acc = 83, F1-score = 84.29, Pre = 87.01, Rec = 83<br>Acc = 89, F1-score = 89.43, Pre = 90.40, Rec = 89
**Our**	2DCNN-GRU	Acc = 97.75, F1-score = 97.75, Pre = 97.75, Rec = 97.75, Spec = 99.25

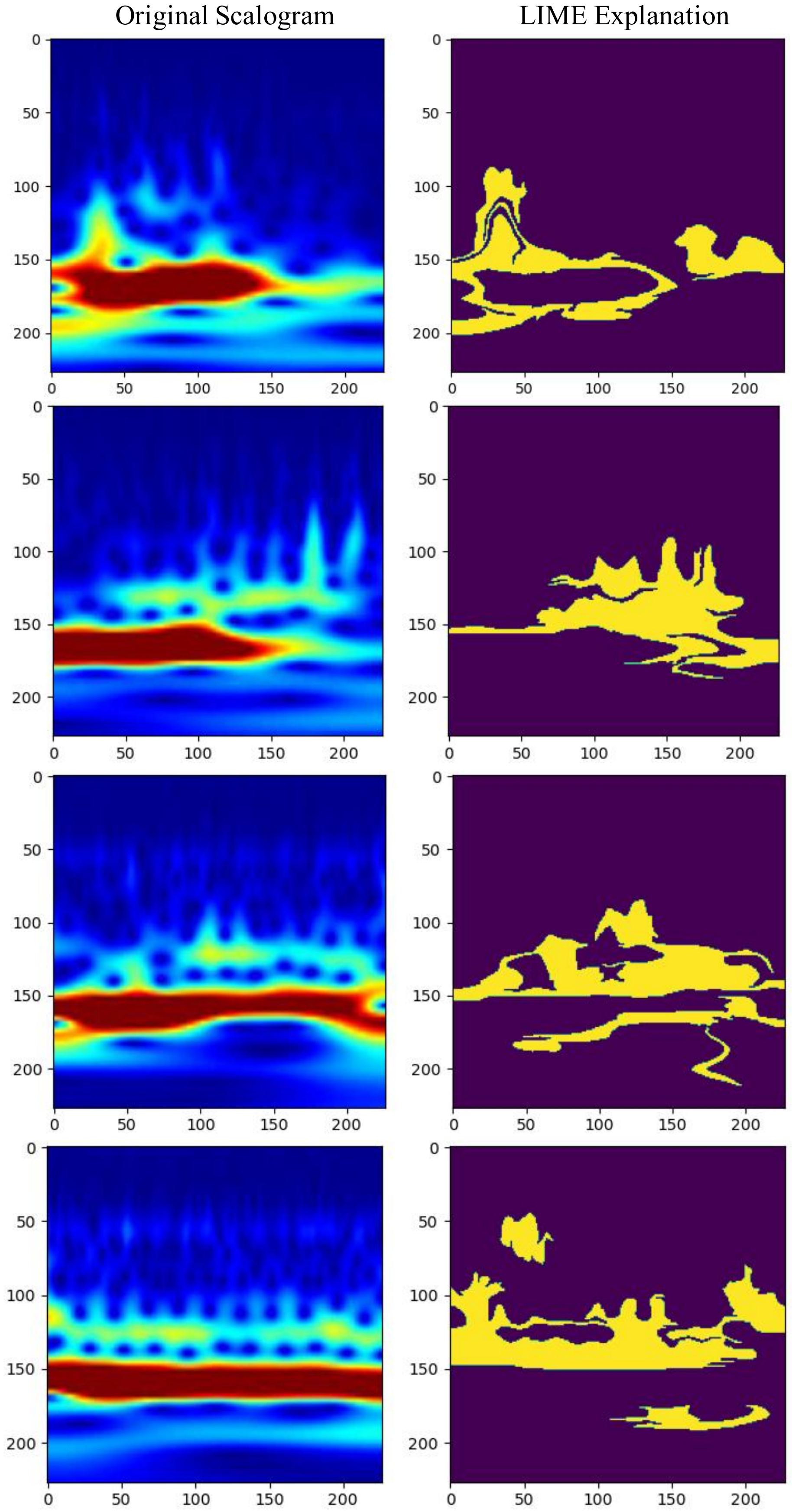

Supplementary Figure S2 | Illustration of interpretation of model predictions using the LIME method.

The left panel shows the original scalogram representing the ECG signal in the time-frequency domain. In contrast, the right panel illustrates the LIME explanation, highlighting the most significant regions influencing the model's prediction.
